# The Frequency of Autoimmune Thyroid Disease in Alopecia Areata and Vitiligo Patients

**DOI:** 10.1155/2015/435947

**Published:** 2015-08-18

**Authors:** Gulcan Saylam Kurtipek, Fatma Gökşin Cihan, Şule Erayman Demirbaş, Arzu Ataseven

**Affiliations:** ^1^Department of Dermatology, Konya Training and Research Hospital, 42090 Konya, Turkey; ^2^Department of Family Medicine, Meram Faculty of Medicine, Necmettin Erbakan University, 42090 Konya, Turkey; ^3^Department of Family Medicine, Konya Training and Research Hospital, 42090 Konya, Turkey

## Abstract

*Aim*. Many studies demonstrated that alopecia areata (AA) and vitiligo are commonly associated with autoimmune thyroid diseases. We aimed to investigate the frequency of thyroid dysfunctions and autoimmunity related with vitiligo and AA.* Material and Methods*. 200 patients, 92 AA and 108 vitiligo diagnosed, were surveyed retrospectively. The control population was in reference range and from Konya, central Anatolian region of Turkey. Thyroid function tests (free T_3_, free T_4_, and TSH) and serum thyroid autoantibody (anti-TG, anti-TPO) levels were evaluated in all patients.* Results*. In vitiligo patients, 9 (8.3%) had elevated anti-TG levels and 16 (14.8%) had elevated anti-TPO, and in 17 patients (15.7%) TSH levels were elevated and 3 (2.8%) patients had elevated fT_4_ levels and 5 (4.6%) had elevated fT_3_ levels. Within AA patients, 2 (2.2%) had anti-TG elevation and 13 (14.1%) had anti-TPO elevation, in 7 patients (7.6%) TSH were elevated, and in 1 patient (1.1%) fT_4_ were elevated and 5 (5.4%) patients had elevated fT_3_ levels.* Conclusion*. In our study, impaired thyroid functions and thyroid autoantibodies in vitiligo and AA patients were identified at lower rates than the previous studies. According to results of this study there is no need for detailed examination in alopecia areata and vitiligo patients without clinical history.

## 1. Introduction

Alopecia areata (AA) is an inflammatory disease characterized by scarless alopecia of the scalp and/or whole body hair. It affects 1% of the general population and this complaint takes up to 0.7–4% of all patients presented to the dermatology clinics [1].

Vitiligo is an acquired, idiopathic disease with no clear etiology and characterized by destruction of melanocyte, which results in development of depigmented macules [2]. Even though the etiology of these two diseases is not fully understood, concurrence of autoimmune diseases and endocrine dysfunction gives rise to the possibility of autoimmune origin.

## 2. Material and Methods

After local ethical committee approval for our study, the records of 92 AA and 108 vitiligo patients who presented to our dermatology clinic between January 2010 and January 2014 were, respectively, reviewed. Thyroid function tests (free T_3_, free T_4_, and thyroid stimulating hormone) and serum thyroid autoantibody (anti-thyroglobulin (anti-TG) and anti-thyroid peroxidase (anti-TPO)) levels of 200 patients were also examined in the study. The AA and vitiligo patient's laboratory data was compared with the reference controls for all the parameters. The normal ranges were according to the laboratory standards of Konya Research and Training Hospital, a reference hospital in Turkey. fT_3_, fT_4_, insulin, and TSH were measured by Advia Cetaur XP with chemiluminescence method (Siemens Healthcare Diagnostics). Anti-TG and anti-TPO levels were measured by Immulite 2000 with chemiluminescence method (Siemens Healthcare Diagnostics). The results obtained from the patients were evaluated based on the reference value set by the manufacturer.

Statistical analysis was done using SPSS 18.0 program. For descriptive analyses, mean, median, minimum, maximum, and standard deviation were used, and to test significancy chi-square test was used. The level of significance was assumed as *p* < 0.05.

## 3. Results

A total of 108 vitiligo patients and 92 AA patients were included in the study. Mean age of vitiligo patients was 30.15 ± 17.07 years and 49.1% of patients were males (*n* = 53) and 50.9% were females (*n* = 55). Mean age of the patients with alopecia areata was 26.25 ± 12.37 years; 59.8% of the patients were males (*n* = 55); 40.2% of patients were females (*n* = 37). Mean free T_3_ (fT_3_) levels were 3.63 ± 0.65 pg/mL in the patients with vitiligo and were 3.67 ± 0.61 pg/mL in the patients with alopecia. Mean free T_4_ (fT_4_) levels were 1.25 ± 0.31 ng/dL in the patients with vitiligo and were 1.32 ± 0.28 ng/dL in the patients with alopecia. While geometric mean of TSH levels was 2.06 (0.01–150.00) micro-IU/mL in vitiligo patients, it was measured as 1.94 (0.45–4.91) micro-IU/mL in alopecia patients. In the patients with vitiligo, geometric mean of anti-TPO levels was 18.52 (7.94–1000.00) IU/mL and in alopecia patient the mean was found as 18.70 (7.38–1000.00) IU/mL. Geometric mean of anti-TG levels was 23.90 (10.00–3000.00) IU/mL in vitiligo patients and the geometric mean was calculated as 21.07 (10.00–3000.00) IU/mL in the patients with alopecia.

Normal values for thyroid function and thyroid antibody tests were defined as fT_3_: 1.57–4.71 pg/mL, fT_4_: 0.85–1.78 ng/dL, TSH: 0.4–4 micro-IU/mL, anti-TPO: 0–35 IU/mL, and anti-TG: 0–40 IU/mL based on the reference value set by the manufacturer; and any values lower or higher than these were accepted as decreased or increased hormonal levels. When the classification is made according to these levels, in 84.3% (*n* = 91) of vitiligo patients, TSH levels were within normal limits, and in 15.7% (*n* = 17) of vitiligo patients the level was higher than normal values. While TSH measurements were evaluated as within the normal limits in 92.4% (*n* = 85) of alopecia patients, it increased in 7.8% (*n* = 7) of the patients diagnosed with alopecia. On the other hand, normal fT_3_ levels were shown in 95.4% (*n* = 103) of the vitiligo patients and in 4.6% (*n* = 5) of these patients the level was high. The levels of fT_3_ were evaluated as normal in 94.6% (*n* = 87), while it was increased in 5.4% (*n* = 5) of the patients with alopecia. fT_4_ levels were within normal limits in 98.2% (*n* = 105) of the patients with vitiligo and were high in 2.8% (*n* = 3). fT_4_ measurement was normal in 98.9% (*n* = 91) of the patients with alopecia and it was high in 1.1% (*n* = 1) (Table 1). Evaluation of anti-TPO and anti-TGA levels based on the diagnosis of the patients was shown in Table 2. No statistically significant correlation between the gender of the patients and the diagnosis, thyroid function test, and thyroid autoantibody levels could be detected (*p* > 0.05) (Tables 3 and 4).

## 4. Discussion

AA is a disease with unknown etiology characterized by oval or round scarless hair loss. The lesion can be single and self-limiting or may be widespread, and recurrence can be seen in the course of the disease. Its etiology is not fully understood, although genetic susceptibility, autoimmunity, and stress are suggested as some of the causative factors. Some researchers claimed that cell-mediated immunity plays a role on lymphocytic infiltration surrounding anagen hair follicles. Humoral immunity has been also suggested to have an important role in the development of AA. Specific IgG antibody to the hair follicles was found in higher concentration in peripheral blood of the patients with AA compared to control subjects [3–5].

Vitiligo is considered as an autoimmune disease characterized by wide spread white patches which result from loss of melanocytes. Autoimmune diseases, genetic factors, autotoxic metabolites which occurred during melanin synthesis, accumulation of neurochemical substances, free radicals, deficiency of melanocytes growth factors, and deterioration in function or structure of melanocytes are believed to be responsible factors in the pathogenesis of vitiligo [6].

AA and vitiligo are often observed with other autoimmune diseases. Among these diseases, vitiligo, lichen planus, lichen sclerosus et atrophicus, pernicious anemia, atopic dermatitis, Hashimoto's thyroiditis, hypothyroidism, endemic goiter, diabetes mellitus, lupus erythematosus, and Graves' disease can be included. However, the chance of coexistence with autoimmune thyroid diseases is higher than the others [7–12].

Previous studies which investigated frequency of autoimmune thyroid disease in vitiligo patients revealed very different results. Positive anti-TG titers were found in 30%, and thyroid dysfunction was found in 10–20% of the patients with AA. Likewise, positive thyroid autoantibodies were shown in 2–50% of vitiligo patients. These variable results were believed to stem from possible presence of methodological mistakes and the differences in strength level of the studies [13].

Bakry et al. reported that subclinical hypothyroidism was found in 16% of 50 AA patients and anti-TG antibodies were positive in 23 (46%) of these patients. They also demonstrated positive TPO-Ab titers in 24 (48%) patients [13]. In our study, high anti-TG level was found in 2 (2.2%) patients and high anti-TPO level was found in 13 (14.1%) patients from AA group. Our results were significantly lower compared to the study of Bakry et al. [14].

Abnormalities in thyroid function were demonstrated in 8 (11.4%) out of 70 AA patients in a retrospective study. Significantly high thyroid autoantibody level was present in 18 (25.7%) patients with AA compared to the controls. No significant correlation between presence of antibodies and disease severity was demonstrated [15].

A study regarding 87 vitiligo patients demonstrated positive anti-TG titers in 23% (20/87) of the patients and TPO-Ab were positive in 24.1% (21/87) of the patients, and the results were found significantly higher when compared to healthy controls. Same researchers demonstrated that presence of antibodies was more frequent in female patients between the ages of 11 and 20 [16].

Study of Kasumagic-Halilovic et al. found high levels of anti-TPO in 11 (27%) out of 40 vitiligo patients and they demonstrated significantly elevated levels of anti-TPO compared to the control group [8]. Elevated anti-TPO antibody was present in 14.8% of our patients. Again, Kasumagic-Halilovic et al. investigated frequency of autoimmune disease in 33 vitiligo patients and found thyroid function abnormalities in 6 (18.2%), positive anti-TG antibodies in 9 (27.3%), and anti-TPO antibodies in 8 (24.2%) patients. In the control group, one patient (3.03%) had thyroid hormone abnormality and 2 patients had thyroid autoantibody. They found higher frequency of hormonal abnormality in the patient group compared to our study [17].

Uncu et al. did not demonstrate hypo- or hyperthyroidism in the assessment of 50 children with vitiligo; however they reported significantly higher frequency of autoimmune thyroiditis in girls and in the patients with long duration of generalized vitiligo [18]. In our study we could not find a statistically significant association between the levels of thyroid autoantibodies and the gender.

The study of Ağırgöl et al. regarding thyroid autoimmunity in 112 AA patients and 62 control subjects revealed the presence of autoimmune thyroid disease in 19 (17%) patients and in 3 (5%) control subjects, and the difference between the groups was considered as significant (*p* = 0.015) [19].

Gönül et al. retrospectively reviewed 109-110 AA patients and showed abnormal thyroid function tests in 11 out of 110 (10%) patients and the presence of thyroid autoantibody in 16 out of 109 (14.7%) patients. They demonstrated significant correlation between the duration of AA and the level of autoantibodies [20]. These results are consistent with our results.

In a retrospective study regarding 89 patients with AA, Doğan et al. found Hashimoto's thyroiditis in 5 (5.6%) patients who have been considered as euthyroid; 27% (*n* = 24) of the patients had thyroid function test (TFT) abnormalities. Of these abnormalities, 24.7% (*n* = 22) were considered as clinically insignificant. Anti-thyroid peroxidase antibody (anti-TPO) was found positive in 9% (*n* = 8) of the patients. High levels of anti-TG were present in 3.3% (*n* = 3) of the patients, and these patients have been evaluated as euthyroid. Therefore, they concluded that, regardless of TFT and basal level of thyroid autoantibodies, regular monitoring might be meaningful in children with AA [21]. Autoantibody ratios in that study are similar to those of our study.

In our study, thyroid dysfunction and the level of autoantibodies were lower compared to previous studies. Contrary to some studies, no significant correlation could be observed between the gender and anti-TPO level in our study.

## 5. Conclusion

In our study, thyroid dysfunction and autoantibody level were lower than previous studies. In the lights of present data, we concluded that because of high cost and time consuming nature of the tests detailed thyroid autoimmunity tests are not required for all of the patients with vitiligo and AA who presented to the clinics; we think it is more suitable that further evaluation should be done in the patients with positive family history, if suspicious findings are obtained during the examination.

## Figures and Tables

**Table 1:** Levels thyroid function tests based on diagnosis.

	Normal	High
	*n* (%)	*n* (%)
Vitiligo		
fT_3_	103 (95.4)	5 (4.6)
fT_4_	105 (97.2)	3 (2.8)
TSH	91 (84.3)	17 (15.7)
AA		
fT_3_	87 (94.6)	5 (5.4)
fT_4_	91 (98.9)	1 (1.1)
TSH	85 (92.4)	7 (7.6)

**Table 2:** Levels of thyroid autoantibodies based on diagnosis.

	Normal	High
	*n* (%)	*n* (%)
Vitiligo		
Anti-TG	99 (91.7)	9 (8.3)
Anti-TPO	92 (85.2)	16 (14.8)
AA		
Anti-TG	90 (97.8)	2 (2.2)
Anti-TPO	79 (85.9)	13 (14.1)

**Table 3:** Correlation between gender and anti-TPO levels.

	Female	Male	*χ* ^2^	*p*
	*n* (%)	*n* (%)		
Vitiligo				
Anti-TPO normal	45 (81.8)	47 (88.7)	0.537	0.464
Anti-TPO high	10 (18.2)	6 (11.3)		
AA				
Anti-TPO normal	31 (83.8)	48 (87.3)	0.028	0.868
Anti-TPO high	6 (16.2)	7 (12.7)		

**Table 4:** Correlation between gender and anti-TG levels.

	Female	Male	*χ* ^2^	*p*
	*n* (%)	*n* (%)		
Vitiligo				
Anti-TG normal	49 (89.1)	50 (94.3)	0.992	0.319
Anti-TG high	6 (10.9)	3 (5.7)		
AA				
Anti-TG normal	36 (97.3)	54 (98.2)	0.080	0.778
Anti-TG high	1 (2.7)	1 (1.8)		
